# “A becoming in the meeting”: the interpretations of competence in home care from the perspectives of older people and registered nurses – a meta-ethnography

**DOI:** 10.1080/17482631.2023.2262170

**Published:** 2023-09-29

**Authors:** Karoline Lang Mathiesen, Elisabeth Lindberg, kristina Nässén, Fiona Cowdell, Lina Palmér

**Affiliations:** a Faculty of Caring Science, Work Life and Social Welfare, Doctoral student, University of Borås, Borås, Sweden; b Faculty of Caring Science, Work Life and Social Welfare, University of Borås,Borås Sweden; c Faculty of Caring Science, Work Life and Social Welfare, Senior lecturer, University of Borås, Borås Sweden; d Professor of Nursing and Health Research and NIHR Knowledge Mobilisation Research Fellow, Birmingham City University, Birmingham UK; e Faculty of Caring Science, Work Life and Social Welfare, University of Borås, Borås, Sweden

**Keywords:** Competence, care, caring relationship, dignity, home care, meta-ethnography, older people, registered nurses, temporality, togetherness

## Abstract

**Aim:**

The aim of this meta-ethnography was to identify and synthesize qualitative studies focusing on older people’s and registered nurses’ interpretations of competence in home care.

**Methods:**

The meta-ethnography followed the six phases developed by Noblit and Hare (1988).

**Results:**

In Phase 6, the translation process of the included studies, three themes were identified: i) temporality—the feeling of being of value; ii) dignity—a person, not just a patient; and iii) mutuality of being—togetherness. A synthesis was developed, and the phrase “a becoming in the meeting” emerged.

**Conclusion:**

The sense of becoming includes progress, which means becoming something other than before in relation with others and refers to what constitutes the meeting between the older person and the registered nurse working in home care. Competence originates from becoming in the meeting, and registered nurses should therefore value what they do and hold on to this aspect of caring competence that centres on a caring relationship. It is important for registered nurses working in home care to be able to cultivate a caring relationship.

## Introduction

Ageing in place is a common policy to address rising costs and meet the need and preference of older people to remain in their home in many Western societies (Haex et al., 2020). There is an increasing need for high‐quality, competent home care services and quality measures (Haex et al., 2020), but at present, we do not know what this means to the older persons themselves. A general assumption is that in most current health care systems, the primary understanding of the patient receiving home care is mostly biomedical. This means that curing a physical disease and/or abnormality takes precedence over promoting health as a resource for everyday living, encompassing client choice together with the ability to realize goals and to gain a sense of control in one’s life (From et al., 2013; Haavisto et al., 2020; Turpin et al., 2012).

In terms of understanding competence in older people’s care, the approach of humanization is an important aspect of developing dignified care. Increasing specialization, alongside technological advances and research, has improved health and well-being in home care. However, within that improvement exists a tendency to forget the human dimensions of illness and healing (Todres et al., 2009). The term humanization of care describes an approach to health care that is informed by the core dimensions of what it means to be human (Borbasi et al., 2012). Furthermore, the humanizing care theory aims to understand what matters to people and assess how the process can improve the human dimensions of health care service in the future (Galvin et al., 2020). Human beings are often exposed to both categorization and stigmatization within health care systems (Birdges et al., 2020; Borbasi et al., 2012; Todres et al., 2009). Categorizing people as sick or healthy, or defining what is normal and what is abnormal, also differs from how the patient is conceived from a caring science perspective (Arman et al., 2015, p. 291). The lifeworld perspective reveals an opportunity to develop care that focuses on older people’s perspectives and lived experiences to strengthen health and well-being (Dahlberg et al., 2008). To further strengthen health and well-being among older people, the lifeworld-led caring science approach includes readiness for a caring dialogue that focuses on not only physical or social issues but also existential issues about what it means to be human and to be cared for (Palmér et al., 2020). Well-being should be understood as a person’s experiences of feeling well and being able to do things in life that matter to them. Within such care, the older person is viewed as a human being living a meaningful existence in which temporality, embodiment, intersubjectivity and spatiality are intertwined within the lifeworld (Dahlberg et al., 2008). In many ways, home care facilitates a meeting between a registered nurse and an older person, and the interaction that occurs in the meeting is a fundamental part that needs to be integrated into nursing care and home care (Dahlberg et al., 2008). When referring to *home care*, this review focuses on home health care provided by registered nurses working in home care for older persons, not to be confused with *home help*, which is often provided by health care staff other than registered nurses. The registered nurses’ health care tasks include, for example, sorting and dosing medicine, wound care and other tasks related to the health and well-being of the older person. Still, a registered nurse working in someone’s home needs to pay attention to the holistic perspective of care, including social perspectives and relationships, rather than focusing only on health factors (Dostálová et al., 2021; Jarling et al., 2022).

Extant literature on health care competence focuses on practical caregiving, the ability to put educational knowledge into practice and experience (From et al., 2013; Hupkens et al., 2020; Karlstedt et al., 2015). According to the World Health Organization (2017), health in older age should not be defined by the absence of disease. People worldwide are living longer. Today, most people can expect to live into their sixties and beyond. Every country in the world is experiencing growth in the proportion of older persons in the population. Strong public policies are needed to ensure that positive trends can be sustained and that the benefits of a healthier life can extend to everyone, regardless of where they live or their socioeconomic status (WHO Europe, 2020). As the population ages, more people need complex care within overburdened European care systems. Additionally, there is pressure on the workforce, and its sustainability is uncertain (WHO Europe, 2020). These problems within care systems, both home-based and institutional, represent a threat to life (Holmberg et al., 2012; Zahran et al., 2016), and substantial evidence points to the added unacceptable suffering amassed through failures of systems in offering dignified care in Europe (Anderberg & Berglund, 2010; Fridth et al., 2015). To further develop home care for older people that strengthens health and well-being in a dignified manner, the present study aimed to identify and synthesize qualitative studies focusing on older people’s and registered nurses’ interpretations of competence in home care.

## Methods

### Design

This study used a meta-ethnography approach with six phases: i) getting started, ii) deciding what is relevant, iii) reading the studies, iv) determining how the studies are related, v) integrating the studies and vi) synthesizing the translations (Noblit & Hare, 1988). The review is reported in accordance with eMERGe guidelines to improve the completeness and clarity of meta-ethnographic reporting (France et al., 2019).

### Data collection and analyses

#### Phase 1: getting started

The aim of this meta-ethnography was to identify and synthesize older people’s and registered nurses’ interpretations of competence in home care to offer new understandings of the concept of competence in home care for older people. Initial searches revealed a paucity of literature on this concept. This search was done by the first author and the university librarian. Synthesizing the interpretations and experiences of both older people and registered nurses enables a deeper understanding of how competence is interpreted, specifically in this growing field of home care. This knowledge may be used in planning and delivering future services.

#### Phase 2: deciding what is relevant

Our focus was on interpretations of competence from the perspectives of older people and registered nurses providing home health care. We (KLM, EL, KN, FC, LP) agreed on the inclusion and exclusion criteria in an iterative manner as the search progressed. The final criteria are summarized in Table 1.

**Table 1.** Inclusion and exclusion criteria.

Inclusion Criteria Perspectives of older people, aged 65 years and over, living in and receiving care in their own homes Perspectives of government-employed registered nurses working in home care for older persons in their own homes that is paid for by taxes Peer-reviewed original qualitative research studies Date of publication: 2011–2021 English language
Exclusion criteria Care for older people taking place at nursing homes, day care centres, hospitals or short rehabilitation programmes Quantitative studies Mixed methods studies

**Figure 1.**
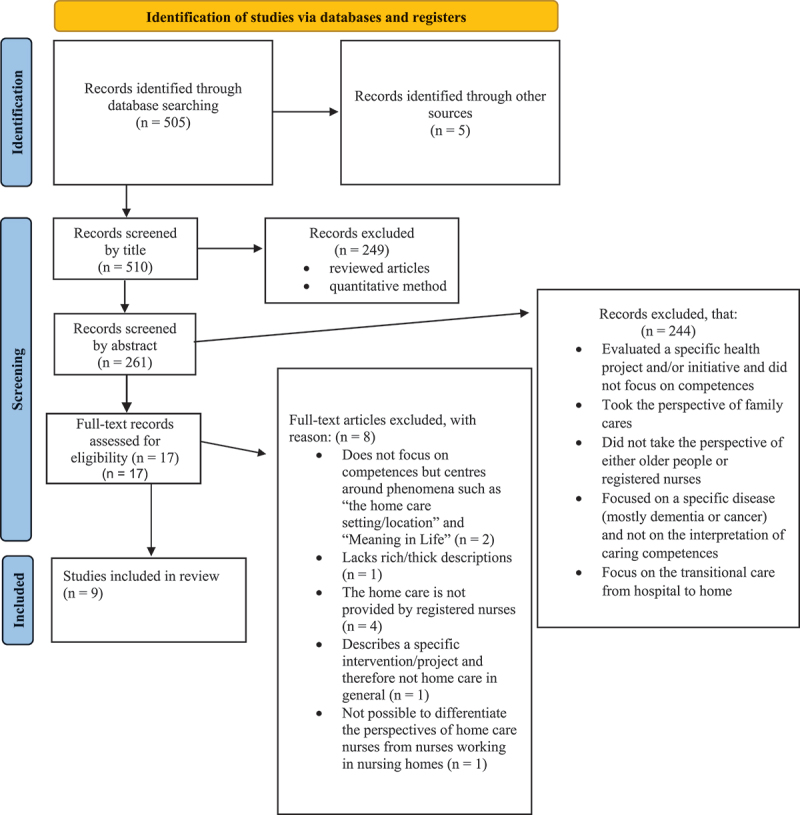
PRISMA flowchart of the literature search.

The CINAHL database was searched using the terms “home care” OR “home health” OR “home nursing” AND skills OR experience OR competenc* AND older OR elderly OR geriatric OR gerontolog×. The limits applied were older people aged 65+ and English language only. Reference searches from the new articles and author searches were conducted simultaneously. The PRISMA search process is summarized in Figure 1. We identified 505 records through CINAHL and 5 records from reference searches of included papers. In total, 488 records were excluded for reasons including using quantitative methods, being reviews and focusing on rehabilitation, hospitals and nursing homes. We read the remaining 17 full-text articles, followed by an appraisal for eligibility and qualification according to the Critical Appraisal Skills Programme criteria for qualitative studies (http://www.casp-uk.net/checklists) (Figure 1). Following discussion, an additional 11 records were excluded for the following reasons:

- Does not focus on competences but centres on phenomena, such as “the home care setting/location” and “meaning in life”.
- Lacks rich/thick descriptions.
- Home care is not provided by registered nurses.
- Describes a specific intervention/project and not home care in general.
- It is not possible to differentiate the perspectives of home care nurses from nurses working in nursing homes.

Eight articles were included in the synthesis (see Table 2). All included studies had sufficient information and thick descriptions to ensure interpretation that may contribute to new knowledge (Noblit & Hare, 1988). Critical appraisal and assigning numerical scores allowed for identification of the “index study”, which is the highest scoring paper based on the validity of the study, the results, whether the methodology is sound and whether the study will influence its field of research (Satter et al., 2021). Our index study is Corbett and Williams (2014).

**Table 2.** List of included studies with study characteristics and metaphors/themes.

Corbett and Williams (2014). Striking a professional balance: interactions between nurses and their older rural patients. **Aim**: To explore the nature and value of the relationship between older adults in chronic pain living in rural areas, and their health and social care providers. **Participants**: Both RN’s (4) and older people (10) **Setting**: home care visits in rural areas of Wales **Method**: Qualitative Interviews and qualitative data analysis **Themes**: Social and emotional connectedness through a familiar face Normal conversation Having time	Haex, R., Thoma‐Lürken, T., Beurskens, A. Haex et al. (2020). How do clients and (In)formal caregivers experience quality of home care? A qualitative approach. **Aim**: To explore and understand the views of clients and formal and informal caregivers about the experienced quality of home care for older people. **Participants**: Both RN’s (4) and older people (6) **Setting**: Dutch home care **Method**: A descriptive qualitative study using content analysis **Themes**: Familiar carers to provide personal care needs Having a connection Attentiveness
Hupkens, S., Goumans, M., Derkx, P., & Hupkens et al. (2020). Nurse’s attunement to patient’s meaning in life—a qualitative study of experiences of Dutch adults ageing in place. **Aim**: To explore the experiences of older adults who receive home nursing, in terms of nurses’ attunement to patients’ MiL **Participants**: Older people (24) **Setting**: home nursing patients in The Netherlands. **Method**: Gadamerian hermeneutic phenomenological design with semi-structured interviews and interpretative phenomenological analysis. **Themes**: Technical skills vs. personalized care Attentiveness Knowing the nurse A special connection	Sæterstrand and Rudolfsson (2019). Using a Reflective Attitude when Meeting Older Chronically Ill Patients’ Care Needs in Home Care Nursing. **Aim**: To describe what nurses take into account in order to meet the care needs of older chronically ill patients in home care nursing. **Participants**: Registered nurses (14) **Setting**: Four different home care units in two municipalities in Norway **Method**: Semi-structured interviews and qualitative content analysis **Themes**: Knowing the patient Listening Making time
Turpin et al. (2012). The meaning of a positive client-nurse relationship for senior home care clients with chronic disease. **Aim**: This study explored the meaning of a positive client-nurse relationship for seniors with chronic disease receiving in-home care. **Participants**: Older people (8) **Setting**: Home care in London, Ontario, Canada. **Method**: Phenomenological study **Themes**: A personal aspect (not just a clinical problem) Knowing the nurse Taking time	Choe et al. (2015). Ethical concerns of visiting nurses caring for older people in the community. **Aim**: The purpose of this study was to explore the ethical concerns that visiting nurses experience when caring for vulnerable older people living in a community. **Participants**: Registered nurses (13) **Setting**: 13 visiting nurses in a South Korean community. **Method**: In-depth interviews and qualitative thematic analysis **Themes**: Time pressure (due to administrative practices) Knowing the patient
Sundler et al. (2020). Attributes of person‐centred communication: A qualitative exploration of communication with older persons in home health care. **Aim**: The aim of this study was to explore attributes of person‐centred communication between nurses and older persons being cared for in their home. **Participants**: Both RN’s (11) and older people (37) **Setting**: Home care in Sweden **Method**: A descriptive study with a qualitative approach and qualitative thematic analysis. **Themes**: Recognizing the person and being familiar Asking questions and listening	Tønnessen et al. (2011). Rationing home-based nursing care: professional ethical implications. **Aim**: The purpose of this study was to investigate nurses’ decisions about priorities in home-based nursing care. **Participants**: Registered nurses (17) **Setting**: Home care in Norway. **Method**: Qualitative research interviews. The interviews were analysed and interpreted according to a hermeneutic methodology. **Themes**: Caring despite time pressure Attention to individual care needs

#### Phase 3: reading the studies

**Figure 2.**
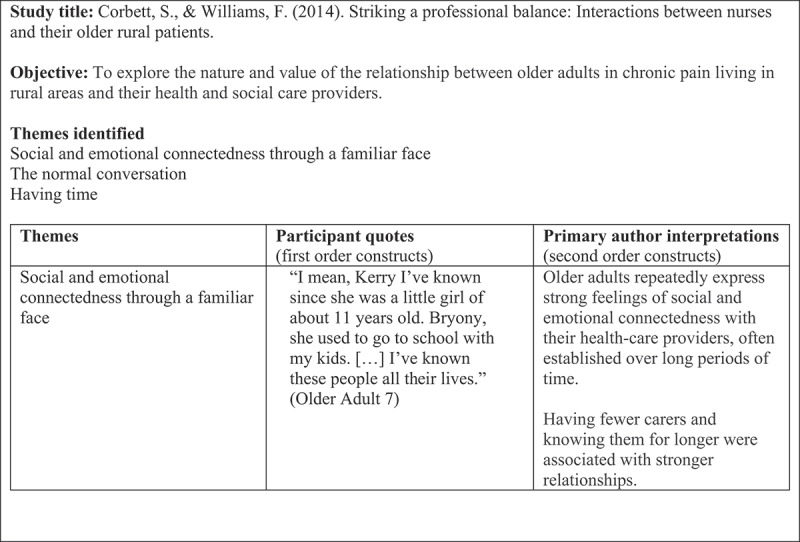
Example of data extraction table.

This stage involved familiarization with the included studies and identification of key concepts in each study. All the studies were read in full to provide context for interpretation and explanation of each study. At this stage, we (KLM, LP) extracted information on study characteristics, including information on the study sample, data collection, analysis methods, study outcomes and conclusions. Once we had read through the chosen studies, we started extracting the “raw data” from the studies for the synthesis. The raw data are the first- and second-order constructs, as exemplified in Figure 2 (Satter et al., 2021, p.5\5). First-order constructs represent the primary data reported in each paper (the participant quotations). The second-order constructs represent the primary author’s interpretations of the primary data (metaphorical themes or concepts). Third-order constructs represent the reviewers’ higher-order interpretations developed from an analysis of the first- and second-order constructs.

#### Phase 4: determining how the studies are related

We (KLM, LP) determined the relationships between studies and key concepts after several readings. Our aim was to identify the core concept, the “meaningful idea that develops by comparing particular instances” (Satter et al., 2021, p. 5). In this phase, we created a list of the themes from each paper (Table 2) and recorded whether the study had included older people (OP), registered nurses (RNs) or both groups. Next, themes from the studies were clustered into categories named using terminology that encompasses all the relevant concepts they contain (Table 3).

**Table 3.** Reducing themes into relevant categories.

**Clustering themes into relevant categories** Taking/making time Having time (Corbett & Williams, 2014; RN & OP) Making time (Sæterstrand T. M., & Rudolfsson, G. 2019, RN) Caring despite time pressure (Tønnessen et al., 2011, RN) Taking time (Turpin et al., 2012, OP) Time pressure due to administrative practices (Choe et al., 2015, RN) Knowing the patient/knowing the nurse Social and emotional connectedness through a familiar face (Corbett,, & Williams, 2014; RN & OP) Familiar carers to provide personal care needs (Haex et al., 2020, RN & OP) Knowing the nurse (Hupkens et al., 2020, OP) Knowing the nurse (Sæterstrand T. M., & Rudolfsson, G. 201; Choe at a;., 2015, RN)9, RN) Recognising the person and being familiar (Sundler et al., 2020, RN & OP) Knowing the nurse (Turpin et al., 2012, OP) Seeing the person, not just the patient The normal conversation (Corbett & Williams, 2014; RN & OP) Attentiveness (Haex et al., 2020, RN & OP) Technical skills vs. personalised care (Hupkens et al., 2020, OP) Attentiveness (Hupkens et al., 2020 , OP) Attention to individual care needs (Tønnessen et al., 2011, RN) A personal aspect – not just a clinical problem (Turpin et al., 2012, OP) Interpretations of connectedness Having a connection (Haex et al., 2020, RN & OP) A special connection (Hupkens et al., 2020 OP) Listening (Sæterstrand & Rudolfsson, G. 2019, RN) Asking questions and listening (Sundler et al., 2020, RN & OP)

#### Phase 5. Integrating the studies

**Figure 3.**
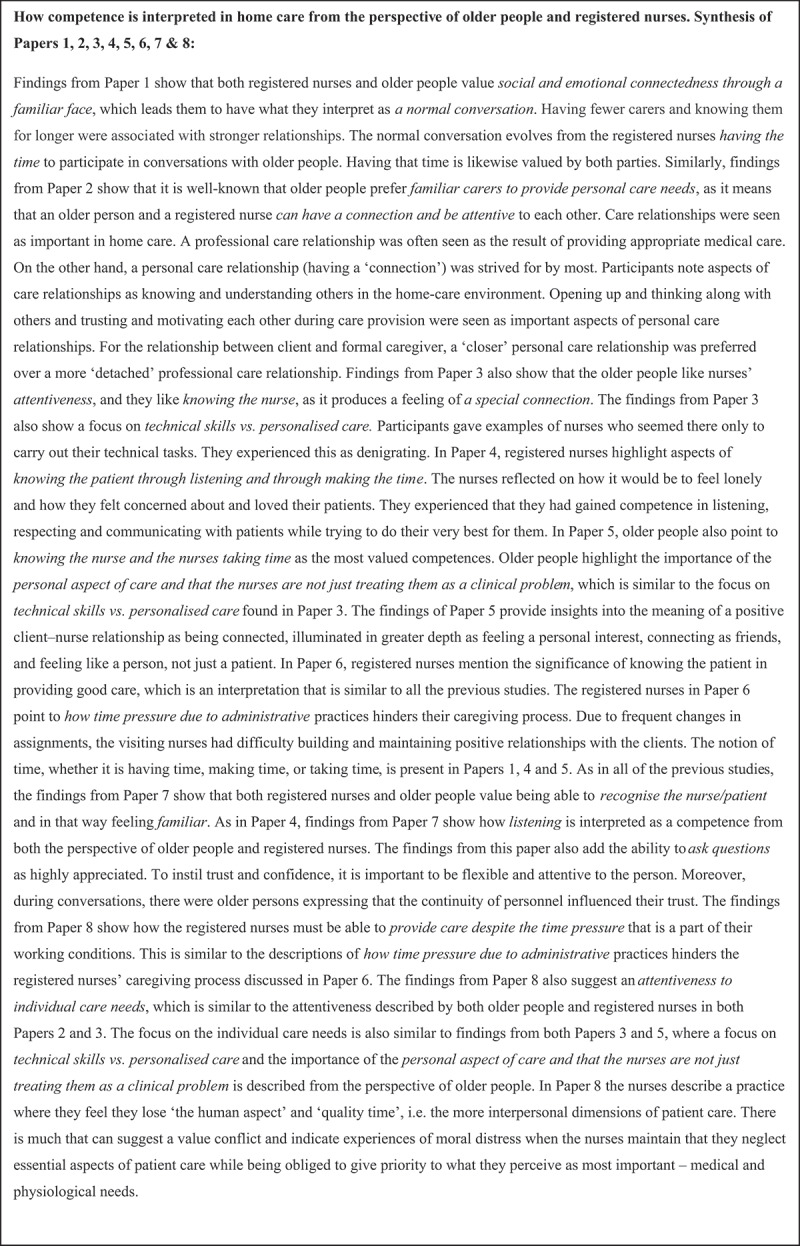
Primary data synthesis of the primary author interpretations.

**Figure 4.**
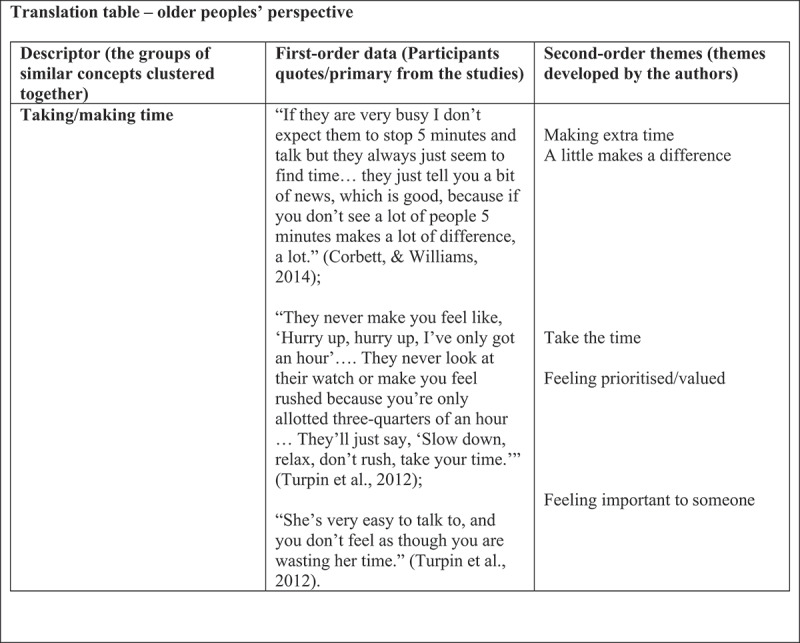
Example of a translation table – older people’s perspectives.

In this phase, each concept was compared across papers to check for the presence or absence of commonality. This highlighted similarities and differences between the concepts and metaphors and allowed us to organize them into further conceptual categories, which resulted in the development of the higher third-order constructs. First, we (KLM, LP) produced a synthesis of the primary author interpretations across papers (Figure 3). We then supported our synthesis by creating a translation table to display this level of synthesis (Figure 4). This process was not linear; instead, we went back and forth between the findings and the primary studies. The translation process involved treating findings as analogies, enabling comparison between similar findings in the various studies (Noblit & Hare, 1988). We compiled two separate translation tables, one for the views of patients and one for the registered nurses’ perspectives (see example in Figure 4).

#### Phase 6: synthesising the translations

During this phase, we (KLM, EL, KN, FC, LP) summarized the shared themes across the studies by placing the first- and second-order constructs side by side to compare them. This led to the generation of new concepts. Our original third-order constructs were further developed by reading the primary data synthesis (Figure 3) alongside the translations table (Figure 4) to draw out the main points and repeated themes (Noblit & Hare, 1988; Satter et al., 2021). Our selected studies were sufficiently similar in their focus to allow for reciprocal translation synthesis. We organized the third-order constructs in a table to enable visual comparison (Table 4).

**Table 4.** Examples of third-order themes.

Third-order themes: Older people studies (interpretation of competence in home care)	Third-order themes: Registered nurses studies (interpretation of competence in home care)
Feeling prioritised/feeling important to someoneImportance of knowing and trusting each otherGetting recognized as a human beingMeaning something to someone Importance of knowing and trusting each other Getting recognized as a human being Meaning something to someone	Making/prioritizing timeBeing familiar/showing interestSeeing/acknowledging the whole person Being familiar/showing interest Seeing/acknowledging the whole person

**Figure 5.**
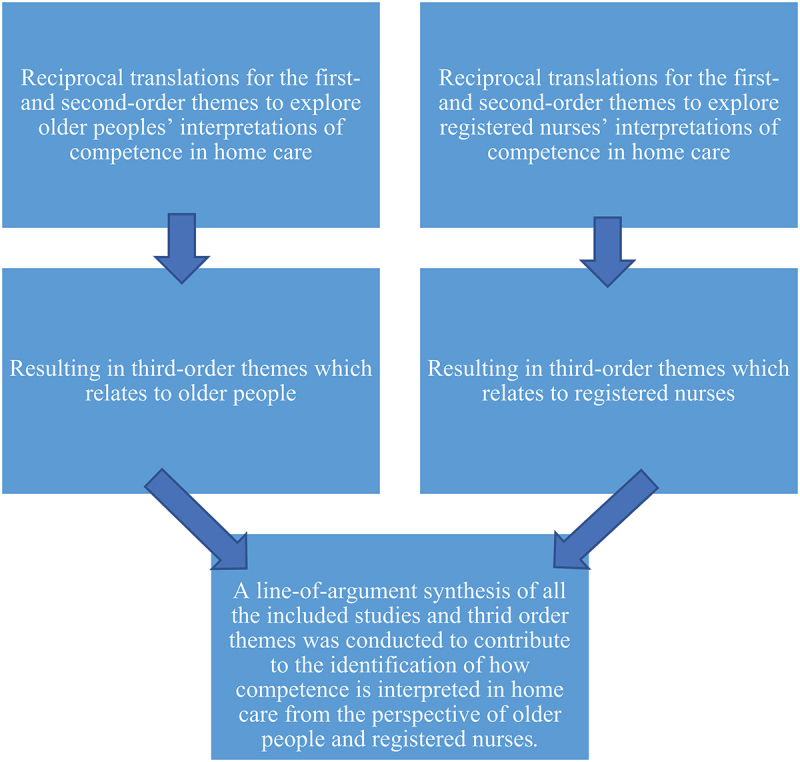
Example of a synthesis process.

**Figure 6.**
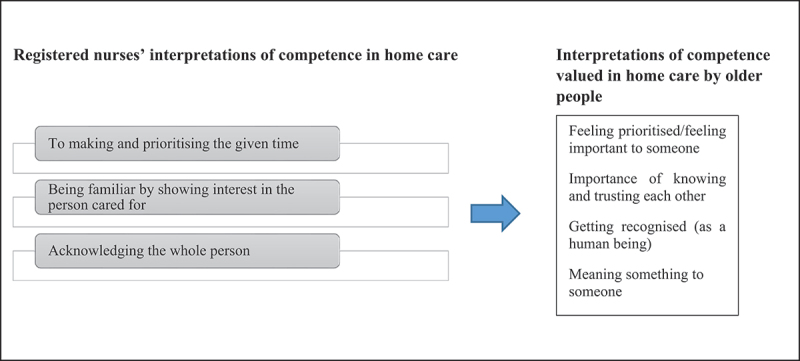
Example of a line-of-argument synthesis developed.

We conducted separate reciprocal translations for the first- and second-order themes relating to older people and registered nurses, resulting in third-order themes that related to one group or the other. The synthesis process comprised three steps (Figure 5). This process resulted in a line-of-argument synthesis (Figure 6). A line of argument became apparent to us during the synthesis, as the concepts from the patient and health care professional studies did not contradict each other; rather, they described different perspectives on the same phenomenon (Satter et al., 2021).

## Ethical considerations

Although this study did not involve human participants, ethical issues were considered in the included studies. We strove to ensure and verify that our interpretation of the findings was based on data reported in the original studies. All original studies stated that they obtained ethical approval from a research ethics committee.

## Results

The eight included papers represent six countries: two each from the Netherlands and Norway and single papers from Wales, Sweden, Canada and South Korea. All of the papers were published between 2011 and 2020. The total number of participants across studies was 148, of which 85 were older people and 63 were registered nurses. Haex et al. (2020) and Tønnessen et al. (2011) did not report on gender, but the distribution in the remaining studies was 38 women and 4 men and 63 women and 22 men for registered nurses and older people, respectively. Information about age was specified in all studies except Haex et al. (2020). The older people were 65–96 years old, whereas the registered nurses were 25–65 years old. The study designs included descriptive, hermeneutic and phenomenological approaches. Data collection was conducted using individual or focus-group interviews, together with observations in some cases. All studies relied on either content analysis, thematic analysis or interpretative analysis guided by phenomenology or hermeneutics. Through the translation process (Phase 6) of the included studies, three themes were identified: i) temporality—the feeling of being of value; ii) dignity—a person, not just a patient; and iii) mutuality of being—togetherness. We discuss the themes in more detail in the following sections.

### Temporality—the feeling of being of value

Temporality includes the human perception, experience and social organization of time, meaning that human beings form social relations through and with the allocation of time. When temporality is interpreted as a competence, it is due to its ability to make connections. From the perspective of an older person, feeling that someone is prioritizing you brings a feeling of being valued and creates a special connection between the older person and the registered nurse (Turpin et al., 2012). This is bidirectional; when, as a registered nurse, you make time for someone, when you prioritize them and experience that mutual feeling of connection, it brings value to you as a person and as a registered nurse (Tønnessen et al., 2011). In short, by the use of temporality, you are able to create value for someone and thereby create value in your own life.The theme of temporality shows itself in various ways. You can take time, make time, waste time, organize time and spend time (Choe et al., 2015; Tønnessen et al., 2011; Turpin et al., 2012), and we talk about quality time, waiting time, allocation of time and extra time (Sæterstrand & Rudolfsson, 2019; Tønnessen et al., 2011). This temporality was captured by one participant as follows: “*It’s the time and stopwatch attitude. It’s difficult to set time. We miss out on a lot now we have to be so bound by time and administrative decisions*” (Tønnessen et al., 2011). At first, time in home care seems to be all about hours, minutes and even seconds (Corbett & Williams, 2014; Tønnessen et al., 2011), but it is mostly concerned with the time that you cannot always count and measure. Temporality sets the scene for the care being carried out. Temporality as a competence interpreted in home care is described in multiple ways, the most common of which is through the concept of quality time. Another aspect of temporality is the way in which it is linked to recognizability, repetition and reappearance: “*Well, to me, it’s really nice if I have somebody I’m very comfortable with, you know? Like after someone has come into your home for 3 or 4 years, you get to know a lot about them. They get to know a lot about you*” (Turpin et al., 2012). In this way, there is a connection between the temporality, the length of time one has known the patient or registered nurse, and a feeling of familiarity. It is only if registered nurses take time with patients, and patients allow the registered nurses to make time, that both get this positive feeling of familiarity (Sæterstrand & Rudolfsson, 2019). Such temporality and an understanding of its importance as a competence in home care allow older people and registered nurses to explore care as a familiar connection that enhances value and being of value to one another.

### Dignity—a person, not just a patient

When dignity is interpreted as a competence by both older people and registered nurses, it is related to a person’s ability to acknowledge another person. This means seeing each other not just as a patient and a professional but first and foremost as fellow human beings. This view of the person is important in both providing and receiving dignified care (Corbett & Williams, 2014; Tønnessen et al., 2011; Turpin et al., 2012). From the registered nurse’s perspective, this means taking the whole person into account by balancing technical skills with emotional support in caring (Corbett & Williams, 2014). Furthermore, registered nurses describe how losing overall perspective can be detrimental in terms of providing care. When registered nurses have to carry out specific tasks in a short time, they are so focused on doing these jobs that they do not see other things—they lose the broad view: “*They see, yet they don’t see. They overlook things and don’t notice major or minor changes or … they do their job and they leave*” (Tønnessen et al., 2011). Other registered nurses describe the importance of not just going in and doing the visit and performing tasks; talking to patients about their lives and their families is part of providing care. This enables patients to talk to you, and you build a relationship: “*It’s part of the treatment, basically, and makes them feel better*” (Corbett & Williams, 2014). Likewise, some older people describe how this feeling of a relationship or the connection with the registered nurses makes them feel better because it makes it clear to them that they are more than just an object receiving care: “*I never felt that I was just a patient. The patient needs to be able to be a person, not a patient. I don’t remember ever being called a patient by these nurses*” (Turpin et al., 2012). Competence in home care dignity can best be described as the mutual acknowledgement of presence that happens in the meeting between a patient and a registered nurse. To the patients and registered nurses, acknowledgement, together with a basic interest in the human being in front of you (Hupkens et al., 2020), is what constitutes dignity as a competence. By acknowledging the whole person and being recognized as a fellow human being, both the registered nurses and the older people feel like care is provided to its fullest (Hupkens et al., 2020; Sæterstrand & Rudolfsson, 2019).

### The mutuality of being—togetherness

When a mutuality of being is interpreted as a competence by both older people and registered nurses, it is related to a feeling of togetherness. A mutuality of being is created in the meeting between an older person and a registered nurse. This means that by being there and being present in the meeting, the registered nurses and older people create a mutual feeling of togetherness, which is why we present this *togetherness* as a competence in home care.

One older person beautifully stated, “*I see the carers as really an anchor to reality. These are ordinary bods out there doing things who bring their world into me*” (Corbett & Williams, 2014). Hereby, the registered nurse and the older person become a part of each other’s life and life stories and create togetherness. Another older person described this togetherness as a feeling of friendship: “*I just want to be human among other humans … There is one nurse who calls me her friend. That’s so nice*” (Hupkens et al., 2020). A registered nurse concluded that by saying, “*We try to get to know the patient by being there. We look at their photos, we talk about children, about travels and about the person’s life*” (Sæterstrand & Rudolfsson, 2019). A patient having the same registered nurse every time may also support a feeling of togetherness because “*the nurse gets used to you and you get to know the nurse*” (Turpin et al., 2012). These are all examples of how togetherness is interpreted as a competence in home care from the perspectives of both registered nurses and older people. We elaborate further on the concept of the *mutuality of being* and its meaning in the interpretation of competence in home care in the following section.

## Synthesis

**Figure 7.**
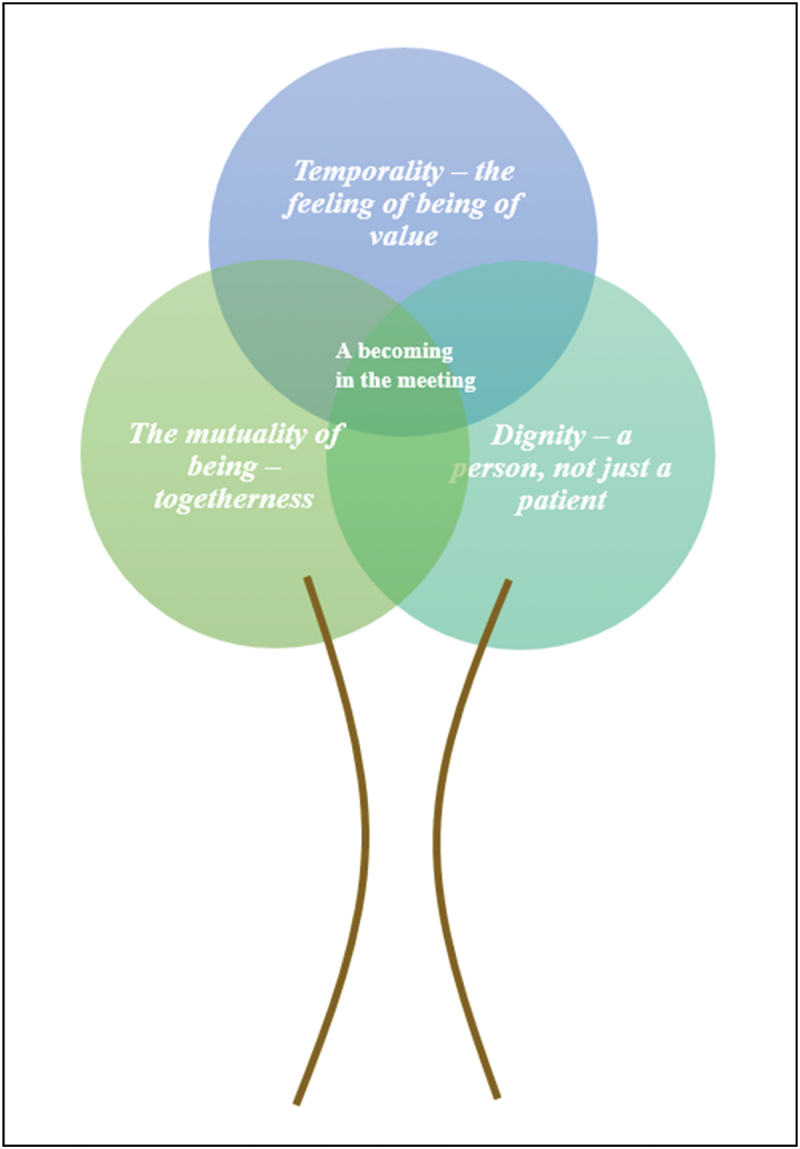
“A becoming in the meeting”.

Competence in home care is interpreted by older people and registered nurses as feelings of being of value, being a person and not just a patient, and togetherness. From these findings, the phrase “A becoming in the meeting” emerged. With a metaphoric model, we aim to show how the interpreted competences all relate back to what is created in the exact meeting between human beings, as illustrated in Figure 7. In this context, *becoming* means *becoming something other than you were before in relation to others*. This means being of value, being prioritized and acknowledged and, most importantly, experiencing a mutual dignified relationship or interest in each other as human beings. A becoming in the meeting symbolizes the importance of acknowledging each other’s presence in home care.

In many ways, home care facilitates a meeting between a registered nurse and an older person. By nature, a home care meeting is forced rather than chosen, which does not always lay the best foundation for a sense of becoming. However, just like a tree needs firm roots, the meeting between a registered nurse and an older person is nurtured not just by a physical presence but by an actual being and togetherness—and that is what constitutes a becoming in the meeting. We elaborate on why competence is something that is often created in a meeting and in collaboration with others in the discussion section.

## Discussion—a becoming in the meeting

The aim of this meta-ethnography was to identify and synthesize older people’s and registered nurses’ interpretations of competence in home care to offer new understandings of the concept of competence in home care for older people. Three analytical themes were identified: i) temporality—the feeling of being of value; ii) dignity—a person, not just a patient; and iii) the mutuality of being—togetherness. These themes highlight different and intertwined aspects of the interpretation of competence in home care from the perspectives of older people and registered nurses and lead to a synthesis described as “a becoming in the meeting”. This means that it all leads back to what occurs in the meeting between the older person and the registered nurse. The sense of becoming includes progress, which means *becoming something other than you were before in relation to others*.

Previous research on competence in home care highlights a combination of knowledge, performance, skill, attitudes and values (From et al., 2013; Haavisto et al., 2020; Karlstedt et al., 2015), but nursing competence in the care of older people is also described as more than these competences; it is also about aspects such as personal insightfulness and motives, interpretive ability and openness towards others (From et al., 2013), which agrees with the findings of this study. This suggests that a combination of education and personal aspects, such as experiences of working to provide care, can influence the competence of a registered nurse (Karlstedt et al., 2015). Following Karlstedt et al. (2015), this means that developing nursing competence is an ongoing process rather than a fixed state. Such an ongoing process is described in our synthesis as a becoming in the meeting.

According to other scholars, the general assumption is that patients’ physical needs are usually prioritized, and emotional and spiritual support of ageing and/or dying patients is lacking, as it may also be more challenging to provide (From et al., 2013; Haavisto et al., 2020). Additionally, registered nurses’ competence in cultural and religious care is often considered to be poor (From et al., 2013; Haavisto et al., 2020). According to Hupkens et al. (2020), care should always be provided adequately and tailored to the individual. These researchers further describe an interpretation of competence in home care through the use of various concepts. One of these concepts is *healthy ageing*, which is considered an important objective for both registered nurses and patients. What healthy ageing actually means is not commonly agreed upon but rather depends on one’s definition and position in the field of health care, as it can refer to both a patients’ physical and mental health (Hupkens et al., 2020). The term *positive health* is regarded as relevant in the care of older people with long-term health problems due to its holistic and subjectivist character, as such patients have to learn to live with these health problems (Hupkens et al., 2020). However, when caring is linked to a written form, it makes it harder for registered nurses to adapt the care to the individual needs of the patient, as they often follow a tight time schedule and must prioritize time for documentation (Sæterstrand & Rudolfsson, 2019).An alternative view offered in the Nordic tradition of caring science has expanded caring abilities by adopting a hermeneutical, phenomenological or lifeworld approach. According to this, competence is obtained by bringing attention to self-awareness and knowledge-based self-development; caregivers tend to be more likely to recognize their patients’ unspoken needs and discover deeper, existential concerns (Arman et al., 2015). With a lifeworld perspective as a basis, Dahlberg et al. (2008) describe caring as grounded in an understanding of the worlds of others. This view on competence is in line with the findings of this study, as it suggests that competence may not be something people carry with them in their backpack of experience and knowledge but rather something that is created in an exact meeting between older people and registered nurses. Here, the context of the meeting shapes how competence is interpreted, regardless of the individuals giving or receiving the care. Competence originates out of *a becoming in the meeting*, as does the interpretation of a relationship between the older person and the registered nurse. The concept of mutuality of being describes the fact that individuals share their lives and existence with each other. In this mutual existence, individuals are described as “persons who participate intrinsically in each other’s existence; they are members of another” (Sahlins, 2013, p. ix). Such human beings move in and out of each other’s lives, perhaps even without actively participating. It is exactly this participation in each other’s existence, whether it is active or not, that creates the feeling of *a becoming in the meeting*. The concept of the mutuality of being includes “the mysterious effectiveness of relationality” (Sahlins, 2013, p. ix). Perhaps it is this mysterious effectiveness of relationality that makes a relationship seem important to both older people and registered nurses working in home care. By entering each other’s lives, and in that way participating in each other’s existence, older people and registered nurses somehow create a mutual feeling of a reciprocal relationship that is highly valued in home care.

## Strengths and limitations

Competence is a rather difficult and complex term to define, so in trying to clarify how it is interpreted in home care from the perspectives of older people and registered nurses, we turned to literature. The database of our choice is the most extensive and comprehensive database of literature on nursing and caring, and by one of the inclusion criteria being qualitative studies, we have already narrowed down the understanding and the complexity of the concept of competence. All the studies focused to some extent on competence in home care, but through the analysis process, we became aware of the complexity of the concept. Sometimes competence is interpreted as related to outcome-specific medical treatments, sometimes it is referred to in connection with efficiency, and sometimes it is viewed as a resource for everyday living, focusing on the patient’s world (From et al., 2013; Haavisto et al., 2020; Turpin et al., 2012). The database of our choice is the most extensive and comprehensive database of literature on nursing and caring, and by one of the inclusion criteria being qualitative studies, we have already narrowed down the understanding and the complexity of the concept of competence, even though the concept is complex and we found some similarities in the included studies.

Our rationale for searching the CINAHL database alone was twofold. One of the strengths of meta-ethnography is that is focuses on identifying a purposive rather than exhaustive data set, which leads to the included papers being the ones that provide the most fruitful data to address the review question, bringing very descriptive interpretations into a review (Doyle, 2003).This is also one of the limitations of meta-ethnography as a scientific method. In focusing on thick descriptions rather than a complete data set, there is a chance of overlooking existing knowledge. In this review, we did not study whether the cases were similar, related or conflicting (Doyle, 2003) but drew on descriptive interpretations of the concept of competence. Analysing the findings from a theoretical perspective and not taking a purely descriptive or thematic approach brings new value to meta-ethnography as a scientific method and to the aim of this review. In the following, we draw on caring sciences theory and anthropology to further develop knowledge about competence in home care for older people. We draw on the Nordic tradition of caring science and its focus on phenomenology and lifeworld theories to describe caring as grounded in an understanding of the worlds of others (Dahlberg et al., 2008). With an anthropological understanding of how individuals share their lives and existence with each other (Sahlins, 2013, p. ix), we aimed to describe how competence is interpreted as a becoming in the meeting and why competence is something that is often created in a meeting and in collaboration with others.

## Conclusion

Competence in home care for older people is an important topic and needs to be discussed. In this meta-ethnography, we identified and synthesizes older people’s and registered nurses’ interpretations of competence in home care to offer new understandings of the concept of competence in home care for older people. The important task for this meta-ethnography was to analyse the findings from a theoretical perspective to contribute new knowledge about the complexity of the concept of competence, in particular bringing new perspectives into the field of home care for older people by viewing it as a becoming in the meeting. Hereby, we hope to start a conversation on how home care for older people could look in the future by implementing this perspective.Ultimately, it all comes down to what happens in that exact meeting between two human beings. The most important thing that brings older people and registered nurses together is to be there in these spaces of possibility. This speaks to not only a physical togetherness but an actual being and presence. In future studies, it will be important for researchers to enlarge the knowledge base of competence from the perspectives of older people and registered nurses and enhance the present understanding of caring competence in home care for older people while integrating these interpretations in nursing care to improve health and well-being for older people. Registered nurses working in home care should themselves value what they do and hold on to this aspect of caring competence that centres on a caring relationship. It is important for registered nurses to be able to cultivate a caring relationship. As our results show, this is done through time that you cannot always count and measure, as temporality is closely linked to the feeling of being of value. Another way to cultivate a caring relationship could be through a sense of togetherness, which means that the registered nurses have to be willing to give a lot of themselves in their job—and this willingness needs to be valued and prioritized to focus on caring relationships as competence in home care.

## Geolocation information

Denmark and Sweden
